# NCBI2RDF: Enabling Full RDF-Based Access to NCBI Databases

**DOI:** 10.1155/2013/983805

**Published:** 2013-07-28

**Authors:** Alberto Anguita, Miguel García-Remesal, Diana de la Iglesia, Victor Maojo

**Affiliations:** Biomedical Informatics Group, Artificial Intelligence Laboratory, School of Computer Science, Universidad Politécnica de Madrid, Campus de Montegancedo S/N, Boadilla del Monte, 28660 Madrid, Spain

## Abstract

RDF has become the standard technology for enabling interoperability among heterogeneous biomedical databases. The NCBI provides access to a large set of life sciences databases through a common interface called Entrez. However, the latter does not provide RDF-based access to such databases, and, therefore, they cannot be integrated with other RDF-compliant databases and accessed via SPARQL query interfaces. This paper presents the NCBI2RDF system, aimed at providing RDF-based access to the complete NCBI data repository. This API creates a virtual endpoint for servicing SPARQL queries over different NCBI repositories and presenting to users the query results in SPARQL results format, thus enabling this data to be integrated and/or stored with other RDF-compliant repositories. SPARQL queries are dynamically resolved, decomposed, and forwarded to the NCBI-provided E-utilities programmatic interface to access the NCBI data. Furthermore, we show how our approach increases the expressiveness of the native NCBI querying system, allowing several databases to be accessed simultaneously. This feature significantly boosts productivity when working with complex queries and saves time and effort to biomedical researchers. Our approach has been validated with a large number of SPARQL queries, thus proving its reliability and enhanced capabilities in biomedical environments.

## 1. Introduction

Over the last decade, there has been a paradigm shift regarding how biomedical data is used for biomedical research, moving from a single database based approach towards an integrative one based on the seamless access and analysis of data from multiple heterogeneous sources. Different technological advances have contributed to this shift in focus, from which two of them stand out over the rest. On one hand, the breakthrough in high-throughput techniques for “omics” data—for example, genomic, proteomic, transcriptomic, epigenomic, cytomic, and so forth—generation has led to the development of novel databases providing a myriad of original data ready to be exploited [1, 2]. On the other hand, advances in telecommunications, improvement of transfer bandwidths, and the increasing ability to access remotely located databases over the Internet have hugely facilitated the access and sharing of biomedical repositories. This way, researchers have gained access to vast amounts of data, enabling them to undertake new lines of research [3–7].

 The benefits of the integrative approach are manifold, mainly in the area of enhanced diagnosis and treatment of diverse diseases [8]. For example, Petrik et al. identified biomarkers for brain tumors by jointly analyzing “omics” data from brain tumor tissue [9]. Zirn et al. employed integrated clinical and genomic data to obtain genetic biomarkers that allow creating a personalized treatment for each patient [10]. More recent examples are described by Ferrara et al., by integrating metabolomic and transcriptional profiling to “construct causal networks for control of specific metabolic processes in liver” [11]. Connor et al. performed integration of metabolomics and transcriptomics data to discover biomarkers related to type 2 diabetes [12]. Elkan-Miller et al. identified several miRNAs functionally important in cases of deafness in mammals after integrating transcriptomics, proteomics, and microRNA analyses [13]. Yi et al. carried out integration of genomic and epigenomic data to “identify key genes and pathways altered in colorectal cancers (CRC),” leading to a prognostic signature in colon cancer [14].

However, the integrative data access approach involves a more complex data handling process. Therefore, researchers and/or database curators will often need carrying out a homogenization and integration step prior to analyzing the data. Heterogeneities among disparate data sources greatly hamper this task. Biomedical researchers demand new data access techniques that relief them from the hassle of handling multiple heterogeneous data sources at a time and that allow them to automatically perform the data homogenization process. To overcome this problem, there have been numerous efforts in the bioinformatics community towards providing methods, tools, and standards aimed at facilitating the integrated access to heterogeneous data sources. One of the most important achievements has been the development of RDF (http://www.w3.org/RDF/), a framework for describing generic resources. RDF was created by the W3C consortium and accepted as a standard in 2004. The development of RDF has facilitated the creation of numerous resources for describing specific knowledge areas. These resources are used in the database integration field as shared vocabularies, providing unified frameworks—that is, shared conceptualizations—that simplify the homogenization process of disparate data [15]. In this sense, ontologies have been established as shared vocabularies [16], generally using RDF itself as the representation language, or any of its extensions (OWL (http://www.w3.org/2004/OWL/), OWL2 (http://www.w3.org/TR/owl2-primer/)). The development of ontologies over the last few years has provided an extensive collection of formal domain descriptions, especially in the area of biomedicine. Some of the most well-known contributions are, for instance, the Gene Ontology (GO), offering a representation of gene and gene product attributes across species [17], the Foundational Model of Anatomy (FMA), built as a symbolic representation of the phenotypic structure of the human body [18], the ACGT Master Ontology (ACGTMO), a thorough description of the area of modern clinical trials on cancer [19], and the Protein Ontology (PRO), a “formal, logically-based classification of specific protein classes including structured representations of protein isoforms, variants, and modified forms” [20]. On top of these developments, there also exist several initiatives targeted at gathering collections of relevant ontologies and providing them publicly. One of these initiatives is the OBO Foundry, a consortium dedicated to the establishment of good practices in ontology development [21]. Another relevant initiative is BioPortal, which “provides access via Web Services and Web browsers to ontologies developed in OWL, RDF, OBO format and Protégé frames” [22].

Regarding state-of-the-art formalisms for querying RDF-based data sources, SPARQL (http://www.w3.org/TR/rdf-sparql-query/) is the most widespread query language at the time of writing this paper. It was developed by the W3C consortium and became a standard in 2008. Nowadays, RDF and SPARQL have been established as the *de facto* data model and query language for representing and accessing biomedical information, respectively. Most biomedical research institutions provide RDF-based access to their repositories, and there are several initiatives targeted at automatically providing RDF views of existing data—for example, Bio2RDF [23]—and SPARQL endpoints to access them. There are also approaches that propose adopting RDF as a solution to increasingly overwhelming sizes of biomedical databases [24]. 

Among the biomedical data sources that researchers often access and use in their work, a very important one is the public set of databases hosted by the National Center for Biotechnology Information (http://www.ncbi.nlm.nih.gov/) (NCBI). The NCBI was founded more than two decades ago with the mission of providing researchers with access to the most relevant biomedical databases. However, and despite its importance, the NCBI data access service lacks the ability to access the data in an RDF-compliant form. In this paper, we present NCBI2RDF, a system designed to provide RDF-based access to the NCBI databases through SPARQL query endpoint. Furthermore, NCBI2RDF offers increased expressivity and enhanced functionalities embedded in its query endpoint, compared to those offered by the native interface of NCBI. 

Next section describes in detail the NCBI database system and its native querying interface.  Section 3 explains our approach for translating and decomposing SPARQL queries into simple queries supported by the NCBI databases. In Section 4, we describe how our system would service a sample query.  Section 5 discusses the benefits of our approach and compares it with other related initiatives. Finally, Section 6 provides a summary and the conclusions.

## 2. Background

The NCBI was established in 1988 with the goal of offering computerized access to a wide set of biomedical data repositories. Its infrastructure has been continuously growing by adding new databases, services, and tools. In 2009, PubMed itself was accessed almost 100 million times each month (http://www.ncbi.nlm.nih.gov/About/tools/restable_stat_pubmed.html). This has pushed the NCBI data sources as one of the most important biomedical data resources for biomedical researchers (http://1degreebio.org/blog/?bid=146/).

The NCBI manages a large set of biomedical databases storing different types of data, all free to access. These include, as of May 2013, over 50 databases ranging from citations and abstracts (PubMed) to genetic repositories (Gene, GenBank, etc.). The background of users accessing these resources includes physicians, biologists, and medical informaticians, bioinformaticians, medical students. In order to provide these users with Internet-based access to the data, the NCBI offers a web-based interface for accessing and displaying the data of their hosted databases. This interface, called Entrez (http://www.ncbi.nlm.nih.gov/sites/gquery/) [25], provides a simple entry point for searching biomedical information based on keyword-based searches—including terms from selected lexical resources such as the MeSH thesaurus—and links among related data.

**Figure 1:**
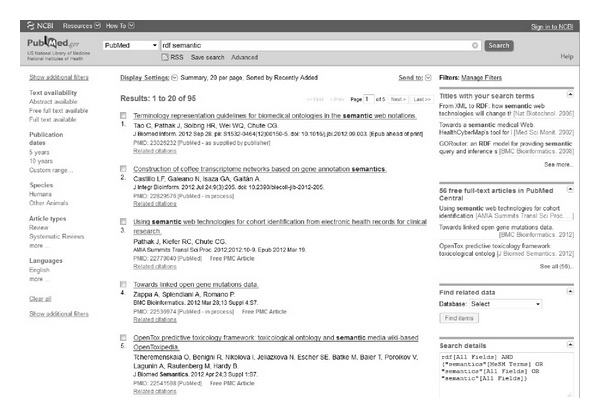
Screen capture of the Entrez system. This screen shows the UIDs of the results for the search of the term “*rdf semantic*” over the PubMed database. For example, the first result shows the UID 23026232.

The Entrez system is focused on ease of use. Its interface features a simple HTML form for specifying search filters over either one of the databases or the entire repository set. This provides users lacking technical background an adequate access point to the stored data. Searches through the Entrez system produce web pages containing a list of UIDs, the global identifier used in NCBI to refer to every entry, independent of the database. Each UID provides a link to an HTML page displaying detailed information about the selected entry—a scientific publication in the case of the PubMed database or a gene description in the case of the Gene database. The inner details of these HTML pages depend on the nature of the queried database. For instance, results from PubMed include details about the retrieved scientific publication—title, journal, authors, abstract, and so forth. These individual result pages may also cross-reference other UIDs which are related to the currently selected result.  Figure 1 depicts a screenshot of the Entrez interface showing the results of the sample query “rdf semantic.”

The Entrez navigation-based system allows nontechnical users to easily access the data stored at the NCBI repositories. The interface allows either performing a general term search in all NCBI databases or defining a more complex query for one specific database. However, the system lacks the ability to enable users to customize the structure of the results—that is, choosing the data fields to be retrieved for each item on the result set—or even to display compound results created by integrating records from different databases. These constraints greatly limit the expressivity of allowed queries in Entrez. Therefore, we believe that Entrez is a suitable interface for performing simple searches, but impractical for more complex situations involving accessing large amounts of interrelated data.

The NCBI offers a second approach for accessing their data: the Entrez Programming Utilities (E-utilities) (http://www.ncbi.nlm.nih.gov/books/NBK25500/). The E-utilities are a set of web-based services where queries are submitted as URLs and results are provided in simple HTML pages or XML documents. The URLs contain all the information needed by the server to resolve the query—database, filters, number of desired results, and so forth— and can be constructed using a simple set of rules. This interface is targeted at developers who intend to build applications that access the NCBI repositories. The E-utilities also implement a powerful feature for reusing results from queries: the history server. The history server maintains recently retrieved lists of UIDs and provides keys for accessing them. The goal is twofold: on one hand, client applications are relieved from repeating queries for accessing frequent data, and on the other hand, the NCBI servers receive a lesser amount of requests. The adoption of the E-utilities by other applications is rather simple. URL codification and result parsing is straightforward. Nevertheless, this is a proprietary format that does not follow any existing standard. In addition, limitations regarding the type of queries allowed with respect to Entrez persist, since queries must be targeted to the whole repository set or to a specific database, excluding any sort of complex query that uses *join* statements.

## 3. Methods

### 3.1. Overview

NCBI2RDF is a Java API for setting up SPARQL-query endpoints over the NCBI databases. It provides a channel for performing SPARQL queries over the NCBI repositories and retrieving data in SPARQL results format. The goal is twofold. On one hand, the API provides RDF-based access to the entire NCBI data, facilitating its integration with other biomedical resources. On the other hand, more powerful queries can be performed compared to the native NCBI querying system, enabling users to launch complex queries involving multiple NCBI databases.

**Figure 2:**
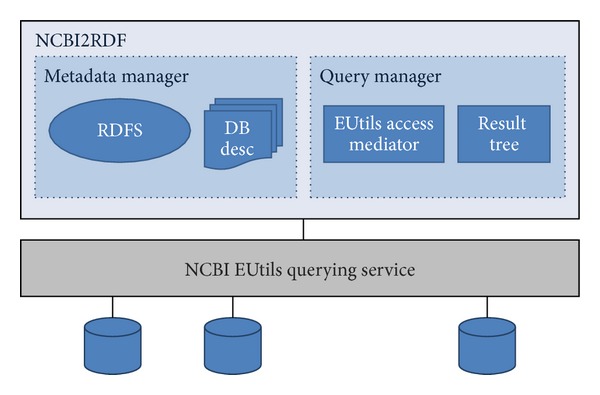
NCBI2RDF system architecture.

NCBI2RDF adopts a dynamic query translation approach for resolving queries. For each SPARQL query posed against the system, NCBI2RDF sets up a workflow of requests to NCBI that allows fetching the data requested by users. This approach stands upon two main processes: metadata generation and query resolution. The metadata generation process consists of building a set of formal descriptions of the data sources available at the NCBI and how to access them. The query resolution process is the one in charge of solving SPARQL queries posed by users.  Figure 2 depicts the system architecture.

The next subsections describe these two processes in detail.

### 3.2. Metadata Generation

The first step in the development of NCI2RDF was obtaining formal descriptions of the databases hosted by the NCBI. NCBI2RDF requires these descriptions to build the RDF schema for the NCBI databases and to be able to access them. The data required by NCBI2RDF includes a list of identifiers of databases stored by the NCBI, a list of fields that each of those databases includes, distinguishing between retrievable fields, that is, those that can be shown within the results, and filterable fields, that is, those that can be used to constrain the searches, and the identifiers of the existing relations between databases. All these descriptions can be obtained through specific web services provided by the NCBI. 

In a first attempt, we decided to generate the needed metadata manually. However, the rather frequent changes that these descriptions undergo forced us to develop an automated process capable of retrieving the repository descriptions and generating the metadata files automatically. This process, called metadata generation, must be triggered regularly to keep the API up to date with the changes in the NCBI databases structure.

**Figure 3:**
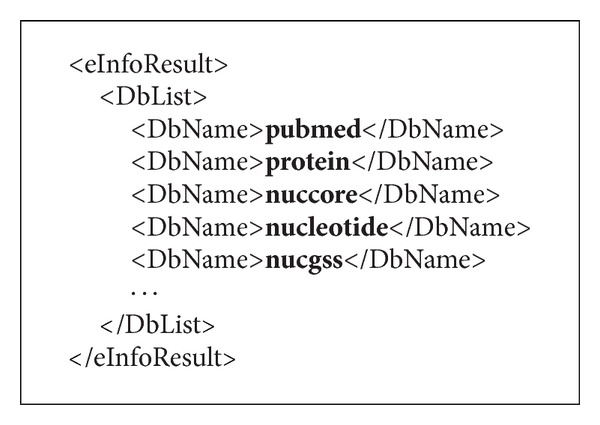
The master XML file listing all available databases.

NCBI provides formal descriptions of all its databases through XML files. Through the web service available at http://eutils.ncbi.nlm.nih.gov/entrez/eutils/einfo.fcgi/, a master file listing all available databases can be retrieved.  Figure 3 shows a code snippet of this master file.

**Figure 4:**
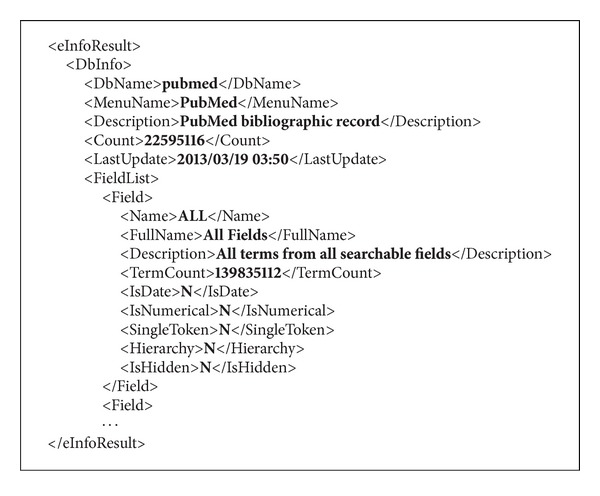
The XML file describing the structure of the PubMed database.

Using the available NCBI database names, it is also possible to retrieve XML files describing each individual database. These XMLs provide general information about the database—that is, a natural language description of the database—and details on its structure, including a thorough description of which fields can be filtered and what relations to other databases are supported.  Figure 4 shows a snippet of the XML file related to the PubMed database, as extracted from http://eutils.ncbi.nlm.nih.gov/entrez/eutils/einfo.fcgi?db=pubmed.

Although these files provide the necessary information about filterable fields and relations between related databases, the list of retrievable cross-linked fields is still missing. The NCBI database management system handles filterable and retrievable fields in a different way, meaning that there exist fields that can be filtered but not retrieved—for example “PDAT”—and vice versa— for example, “LANG.”

The list of retrievable fields is created as follows. For the databases that support the fetch operation—such as PubMed—a related Document Type Definition (DTD) that specifies the retrievable fields can be obtained through a dedicated web service. For any other database which does not support the fetch operation, a valid result document must be manually analyzed for obtaining the final list of retrievable fields. These results are obtained by performing a simple test query.

**Figure 5:**
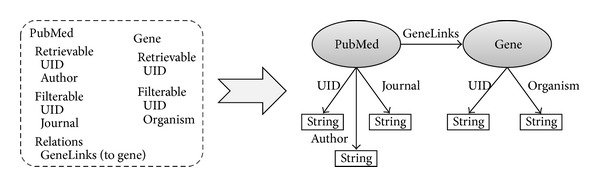
The databases PubMed and Gene in the NCBI repository set produce two related classes in the corresponding RDF model.

Once the list of retrievable and filterable fields and the properties relating different databases has been generated, NCBI2RDF is able to automatically generate the RDF schema that will allow users building correct SPARQL queries. For each NCBI database, an RDF class named after the database is automatically created. Each filterable and/or retrievable field is translated into a *String* datatype in this class. Finally, the generated classes are linked by object properties according to the previously discovered relations between the NCBI databases.  Figure 5 shows a piece of the RDF schema generated from part of the information of the PubMed and the Gene databases.

It must be noted that the asymmetry between retrievable fields and filterable fields is lost when constructing the RDF schema given the inability of this paradigm to model this situation. While our API is able to handle this difference, users will not be able to obtain this information from the RDF schema alone. For this reason, the RDF schema is accompanied with side documentation describing this situation. Users are expected to generate SPARQL queries compliant both with the RDF schema and with the side documentation that forbids that some specific fields are retrieved and other fields are filtered.

### 3.3. Query Resolution

The query resolution process is in charge of accepting SPARQL queries in terms of the previously generated RDF schema and translating them into an equivalent set of requests to the E-utilities services that effectively allow solving the user query. Obviously, SPARQL queries might contain joins between databases—expressed as relations between classes of the RDF schema. As it was described in the background section, NCBI does not allow this type of queries. To overcome this issue, we were forced to develop a solution that would allow us to raise the expressivity of the queries to the desired level, just by using the functions available at the E-utilities services. The solution relies on the simulation of this behavior by means of workflows of simple requests to NCBI that fulfill the original query.

**Figure 6:**
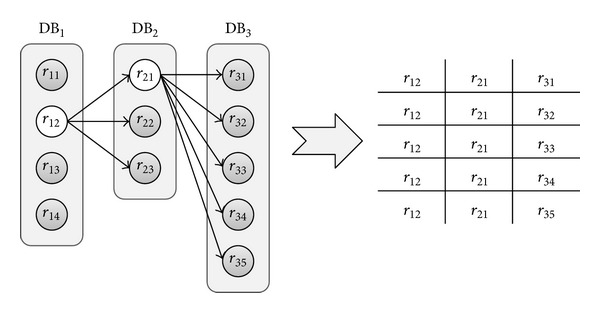
This diagram explains how the tree of results translates into actual results of the queries that the system receives. Each leaf of the tree produces one result, composed of the leaf itself and all the super nodes of that leaf node.

The workflows of requests to NCBI are built in a dynamic fashion. The results of each request help determining the subsequent requests; therefore, it is not possible to assess the complete sequence of requests at once. In essence, each database is queried separately with its own parameters—retrieved variables and filters. Each result produced with this access allows fetching more results from a related database in the query. This process produces what we call “tree of results.” The branches of this tree are built sequentially in a depth-first mode, as each level corresponds to one database in the query. Whenever a branch reaches the last level, results to the original query are gathered from all the different branches that reach from the root node to one leaf. When the results of a branch are exhausted, the algorithm moves backwards in the tree and explores another branch. Figure 6 depicts this process.

The retrieval of each database's results implies several requests to the E-utilities services. As it was described before, these web services provide means to query the NCBI data by external applications. However, complex queries involving more than one database are, as well as with the Entrez interface, not permitted. The operations offered by the E-utilities services are described in the following—only the operations relevant to our query execution process are shown.

- *eSearch* allows retrieving a list of entries of a single database matching some specific criteria. This is the equivalent of the SELECT operation in SQL. The actual result of this operation is not the entries themselves, but the entry identifiers. It is not possible to retrieve entries from more than one database in a single *eSearch* operation—that is, *joins* are not permitted.
- *eFetch/eSummary* allows accessing the information of a single entry from a single database, once the entry identifier has been retrieved using an eSearch operation. The *eFetch* operation retrieves all the fields available for the entry, while the *eSummary* operation offers a subset of this information. Not all NCBI databases support the eFetch operation.
- *eLink* retrieves the list of entry identifiers of a database that are related to a specific entry in another database. This operation resembles the join in SQL. Only one relation can be specified in an *eLink* operation.

Using these operations, a dynamic query resolution workflow is generated. The following algorithm describes this process.

1. The first database in the user query is accessed according to the specified retrievable fields and filters, and its results are stored in the first level of the tree (eSearch + eFetch/eSummary). This is now the current level.
2. If the current level does not correspond to the last database in the sequence, go to 3. Else, the paths from the root of the tree to all the generated leaves form results to the original query. Gather these results and move up one level.
3. Go through the nodes of the current level and, for each node *i*, do:
  - Retrieve the entry identifiers from the next database in the sequence that are related to *i* (*eLink*). These identifiers are stored in a new level hanging from the node *i*.
  - Retrieve the required information for the new nodes (eFetch/eSummary).
  - Make the new level the current one and go to 2
4. Once all nodes were used, prune this branch. If there are levels above the current level, go up one level. Else, finish the data retrieval process.

Once the results are retrieved, they are automatically translated into SPARQL results format and given back to the user. The result returning is performed in a progressive manner, due to the time it requires to completely solve some queries—for example, in queries involving the gene database, there can be millions of results. With our API, clients must request results one by one. To do this, the API offers the typical “iteration” programming schema, with the *hasNext* and *next* methods for exploring the result set.

The constructed workflows are always designed to produce a minimum possible count of requests to the NCBI resources. This helps avoiding the performance penalties imposed for launching multiple requests in a short time lapse. In addition, all accessions to the NCBI databases by means of the E-utilities services are performed using the E-utilities own history service. This allows reducing overload on the NCBI data management system and optimizing performance of our API. 

## 4. Experiments

**Figure 7:**
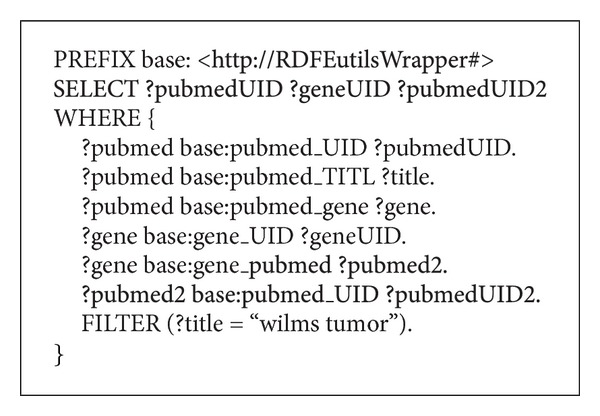
The tested SPARQL query that retrieves articles from PubMed, then related records from Gene, and finally articles that talk about those records from PubMed again.

This section describes how our system deals with an incoming query, providing details of what NCBI-compliant queries are performed to obtain the requested results. The data we intend to retrieve involves three different NCBI databases. We will retrieve papers indexed by PubMed which contain the string “Wilms tumor” in their title. For each of these results, we will retrieve related genes—to Wilms tumor—from the Gene database. Finally, for each of these genes, we will gather again the PubMed database searching for papers that mention that specific gene. Using the NCBI2RDF system, this query can be performed in a single step. The resulting query in SPARQL is depicted in Figure 7.

To process this query, NCBI2RDF automatically generates a dynamic workflow, which is executed as follows. First, a single *eSearch* query targeted at PubMed and including the filter *TITLE=*“*wilms tumor”* is generated, producing 3026 unique results which are stored in the NCBI history server. Then, the first result of this set is retrieved with an *eFetch *query and stored locally—by parsing the XML file representing this result. A third *eLink* query is performed using the recently obtained UID and the *pubmed_gene* relation as arguments, which generates one single result. This result is again retrieved with an *eFetch* query, and a new *eLink* query is realized with the relation *gene_pubmed*. This query returns 557 results from the PubMed database, all of which are fetched simultaneously. The concatenation of the first UID from PubMed, the subsequent UID from Gene, and each of these UIDs composes the first set of retrieved results. To complete the query, the algorithm backtracks twice—since Gene only produces a single result—and selects the second result from the initial PubMed query. This process is repeated until all branches have been fully explored.

Processing a similar query with the NCBI native web interface would involve manually visiting hundreds of thousands of pages. For each of the 3026 initial results in PubMed, the user would have to visit each result individually, and after that, navigate the set of related results in the Gene database. In addition, each Gene result would have to be opened separately, together with its related PubMed records. In the sample query previously shown, our system performed all these steps automatically, producing an average of 50 results per second—note however that this value depends on the complexity of the query. 

## 5. Discussion

The example presented in the previous section shows the advantages that our system presents when compared to the native NCBI interface. The sample query in Section 4 belongs to a test set that we created to validate the system, which included over 200 queries. By raising the expressiveness of the NCBI query processing system, our API enables users to launch complex queries while relieving them from navigating through the NCBI pages that display each result. 

To our knowledge, only the Bio2RDF [23] system is targeted at offering RDF-based access to the NCBI repositories. This system was designed to provide data from multiple biomedical databases in an RDF-compliant form—data can be retrieved in different formats, such as RDF, N3, or plain HTML. However, they base their approach on the manual analysis of HTML documents representing results of queries in order to map its contents to a prefixed RDF structure. This RDF structure was manually created (the Bio2RDF ontology) and only covers some general concepts which are common to all the covered databases. Therefore, Bio2RDF is only capable of providing a few fields for each database. Furthermore, the Bio2RDF interface does not support SPARQL queries and instead resorts to a simple HTML form that allows specifying either single results—through a result ID—or a general search term. This approach allows a quick adoption of new data sources—the system is prepared to be adapted for new relational, HTML, XML, or unstructured databases, but lacks the ability to cover the complexity of each integrated database. Conversely, our approach focuses on maintaining all the information contained in all the NCBI databases and providing SPARQL querying capability over these databases. Moreover, the automated metadata generation procedure permits our system to seamlessly adapt to future changes in the structure of the NCBI databases, or even cover new databases not currently included in the NCBI repositories. Bio2RDF, however, bases its approach on Java Server Pages manually codified for each database, which must be reencoded with each database structure modification.  Table 1 compares the features provided by NCBI2RDF with those of Bio2RDF and the Entrez interface. Conversely, our approach focuses on automatically building a new RDF schema that reflects all databases, relations among these databases, variables, and filterable fields of the NCBI repositories. It is possible, in addition, to define a mapping of this schema to any existing domain ontology—for example, GO, FMA, and PRO—thus enabling the integration of the NCBI data in terms of these vocabularies. The NCBI2RDF approach solves all syntactic heterogeneities between NCBI and the rest of RDF-compliant biomedical data sources. 

This research has been carried out in the context of a large-scale European-funded research project, p-Medicine [26]. This project is aimed at creating a technological infrastructure with data integration capabilities for advanced knowledge discovery in clinical trials in cancer. By integrating the genomic information stored in the NCBI databases within the RDF-enabled p-medicine data infrastructure—which already includes RDF-based access to other relevant sources such as ArrayExpress [27]—we hope to further enhance clinical and genomic information to foster the development of novel personalized drugs for cancer patients based on their genomic profile.

## 6. Conclusions

In this paper, we present NCBI2RDF, an API for providing SPARQL-based access to the NCBI databases. This is achieved by dynamically building native NCBI query workflows. Results from different databases are merged to service complex SPARQL queries involving multiple repositories. The API has been thoroughly tested with a wide range of queries, one of which was presented in Section 4. The presented system effectively provides RDF-based access to all the databases managed by the NCBI. 

Our approach is based on two steps: metadata generation and query resolution. The metadata generation stage gathers information about the structure of the NCBI databases in order to build the subsequent query workflows. This stage is mostly automatic, although some human intervention is still required. However, metadata generation is seldom needed, as the structure of the NCBI databases does not undergo frequent changes. Conversely, the query resolution aims at dynamically constructing the query workflows that will effectively allow servicing SPARQL queries. These workflows emulate the manual work that a researcher would have to carry out in order to retrieve the information distributed across several databases—as explained in Section 4. The workflows are designed to minimize the interaction with the NCBI querying system, in order to save resources.

Our system has two advantages compared to other existing systems—including the NCBI query services themselves. First, the query expressiveness level is raised, since multiple databases can be specified in a single query, saving both time and resources for researchers who wish to perform complex queries against the NCBI system. Second, it enables the semantic integration of the data hosted by the NCBI with other RDF-compliant biomedical resources. 

Current biomedical research is highly dependent on the ability of researchers to uniformly access different data sources—both private and public. However, this capability is mainly hampered by heterogeneities in the data structure, formats, and interfaces. By providing RDF-compliance to the NCBI databases, these heterogeneities are automatically solved, enabling the integrated access of these data with other existing RDF-based repositories. 

## 7. Availability

The software can be freely downloaded as a Java library. Detailed instructions of use are included, as well as complete Javadocs. The project homepage is located at http://www.bioinformatics.org/ncbi2rdf/.

## Figures and Tables

**Table 1:** Comparison of features provided by Entrez, Bio2RDF, and NCBI2RDF for accessing the NCBI databases.

	Query language	Support for complex queries	Support for full NCBI database structure	Access to up-to-date data	Adaptability to changes in NCBI databases
Entrez	HTML form	No	Yes	Yes	Yes
Bio2RDF	HTML form	No	No	No	No
NCBI2RDF	SPARQL	Yes	Yes	Yes	Yes
